# The Modulation of Reward and Habit Systems by Acupuncture in Adolescents with Internet Addiction

**DOI:** 10.1155/2020/7409417

**Published:** 2020-03-13

**Authors:** Yang Wang, Yun Qin, Hui Li, Dezhong Yao, Bo Sun, Zhiliang Li, Xin Li, Yu Dai, Chao Wen, Lingrui Zhang, Chenchen Zhang, Tianmin Zhu, Cheng Luo

**Affiliations:** ^1^School of Acupuncture and Tuina, Chengdu University of Traditional Chinese Medicine, Chengdu, China; ^2^Key Laboratory for Neuroinformation of Ministry of Education, School of Life Science and Technology, University of Electronic Science and Technology of China, Chengdu, China; ^3^School of Medicine, Chengdu University, Chengdu, China; ^4^School of Rehabilitation and Health Preservation, Chengdu University of Traditional Chinese Medicine, Chengdu, China; ^5^Department of Rehabilitation, Zigong Fifth People's Hospital, Zigong, China; ^6^Department of Medicine, Leshan Vocational and Technical College, Leshan, China; ^7^Department of Rehabilitation, TCM Hospital of Longquanyi District, Chengdu, China

## Abstract

**Purpose:**

Acupuncture is an effective therapy for Internet addiction (IA). However, the underlying mechanisms of acupuncture in relieving compulsive Internet use remain unknown. Neuroimaging studies have demonstrated the role of the ventral striatum (VS) in the progress of IA; hence, the aim of this study was to explore the effects of acupuncture on the resting-state functional connectivity (rsFC) and relevant network of VS in IA.

**Methods:**

Twenty-seven IA individuals and 30 demographically matched healthy control subjects (HCs) were recruited in this study. We acquired the functional magnetic resonance imaging (fMRI) data in IA subjects before and after 40 days of acupuncture treatment. Seed-to-voxel and ROI-to-ROI analyses were applied to detect the rsFC alterations of the VS and related network in IA subjects and to investigate the modulation effect of acupuncture on the rsFC.

**Results:**

Compared with HCs, IA subjects exhibited enhanced rsFC of the right ventral rostral putamen (VRP) with the left orbitofrontal cortex (OFC), premotor cortex (PMC), cerebellum, and right ventromedial prefrontal cortex (vmPFC). In the network including these five ROIs, IA also showed increased ROI-to-ROI rsFC. Using a paired *t*-test in IA subjects before and after 40 days of acupuncture, the increased ROI-to-ROI rsFC was decreased (normalized to HC) with acupuncture, including the rsFC of the right VRP with the left OFC, PMC, and cerebellum, and the rsFC of the left cerebellum with the left OFC, PMC, and right vmPFC. Furthermore, the change in rsFC strength between the right VRP and left cerebellum in IA individuals was found positively correlated with the Internet craving alleviation after acupuncture.

**Conclusions:**

These findings verified the modulation effect of acupuncture on functional connectivity of reward and habit systems related to the VS in IA individuals, which might partly represent the underlying mechanisms of acupuncture on IA.

## 1. Introduction

Internet, which improved the ways people live, work, and entertain, has become an integral part of modern life. Despite the great convenience Internet brings, negative consequences have emerged, such as Internet addiction (IA). IA, defined as a behavioral addiction, has become a serious public health issue for its high and fast-growing prevalence around the world [1]. Epidemiologic research among young people has shown that the prevalence rate of IA is as high as 34.4% in some areas [2]. In the last 10 years, the prevalence rate of IA has sharply increased from 3.0% to 26.8% in Hong Kong, with the proportion of heavy Internet users increasing from 25.4% to 42.0% [3].

IA is conceptualized as the compulsive use of Internet with impairments of psychological and social function [4]. Similar with substance dependence, IA is manifested with symptoms such as craving, attentional bias, and compulsive Internet use [5]. Thus, a number of researchers believe that IA might share common neurobiological mechanisms with substance addiction. It has been long known that the striatum plays a pivotal role in substance addiction for the various cognitive functions including reward, motivation, and decision-making [6]. In the widely accepted impaired response inhibition and salience attribution (I-RISA) model, the striatum was associated with 2 of the 6 altered neural systems in substance dependence, which were reward and habit systems [7]. Among the substructures of the striatum, the ventral striatum (VS) was the most relevant to substance abuse, due to the key role in the central dopaminergic nervous pathway [8]. As to IA, accumulating evidence also implicated the morphological and functional changes of VS in progress of this behavioral addiction. According to previous structural MRI studies, altered volumes of the striatum and prefrontal cortex were revealed in Internet addicts, which was correlated with the severity of IA [9, 10]. A task-fMRI study further observed the overactivity of VS in IA individuals when exposed to related cue [11]. Using resting-state fMRI, researchers also found the enhanced functional interaction within VS-frontal circuitry [12], which was implicated as a potential therapeutic target for IA. Collectively, these studies demonstrated the critical role of VS and related circuitry in the development of IA.

A large number of clinical trials have proved the definite effect of acupuncture, a classic therapy in Chinese medicine, on neuropsychiatric disorders including addictions [13, 14]. As a behavioral addiction, IA was also demonstrated responsive to acupuncture [15]. In previous IA studies, acupuncture was found to be effective in alleviating Internet craving [16], attentional impulsiveness [17], and addicted symptoms [18], as well as improving cognitive control function [17]. Nevertheless, little has been known about the neural mechanisms underlying the effect of acupuncture on IA. In our prior magnetic resonance spectroscopy (MRS) study, we observed the increased N-acetyl aspartate and choline levels in the frontal gyrus after acupuncture treatment, implicating the effect of acupuncture in brain neuron protection in IA individuals [17]. However, functional activity was not explored in this study; the modulation of intrinsic brain functional interaction by acupuncture remains unknown, which might provide intuitive insights into the underlying mechanisms of acupuncture on IA.

Resting-state functional connectivity (rsFC), identifying abnormal networks of brain areas exhibiting synchronous blood oxygen level-dependent (BOLD) fluctuations, was extensively used in psychiatric research, including substance addiction [19] and IA [20]. Using rsFC analysis, previous studies have revealed the abnormal functional interaction within the VS-frontal circuit in IA [12] and demonstrated the normalization of the ventral striatal network after a virtual reality treatment in adolescents with IA [21].

Based on the fact that acupuncture is effective for IA, we presumed that acupuncture would modulate the abnormal ventral striatal network in IA. To test our presumption, 30 IA adolescents and 30 matched healthy controls (HCs) were recruited to explore the abnormal rsFC of the VS and related network in IA and the modulation effect of acupuncture. In this study, we attempted to detect the altered rsFC of the VS in IA individuals using seed-to-voxel analysis at baseline. Then, post hoc ROI-to-ROI analysis was performed to explore abnormal rsFC of VS-related network and the modulation of acupuncture on it, selecting the regions that showed altered connectivity to the VS in seed-to-voxel analysis as seeds. Finally, the possible correlations between rsFC changes after treatment and clinical improvement were also assessed.

## 2. Materials and Methods

### 2.1. Participants

All subjects in our study were recruited from college students aged between 18 and 30 years. All subjects were native Chinese speakers and right-handed. The IA individuals fulfilled the diagnostic criteria for IA according to Young's Diagnostic Questionnaire (YDQ) [4]. A score of 50 or higher on Young's Internet addiction test (IAT) was required in IA subjects [22, 23]. On the contrary, the demographically matched HCs were all inconsistent with the IA diagnostic criteria [4]. IAT score was under 50 in HCs. Medical history evaluation and physical examination were conducted to ensure each subject without any other organic diseases or history of substance addiction or mental disorders. Additionally, pregnant or lactating women were also excluded in our study.

All procedures of this study were approved by the Sichuan Regional Ethics Review Committee on traditional Chinese medicine (ethical approval number 2016KL-005), in accordance with the Declaration of Helsinki. All participants signed informed consent forms before enrollment.

### 2.2. Acupuncture Treatment

All IA individuals received standard acupuncture treatment performed by experienced practitioners according to the guidelines of acupuncture. Baihui (DU-20), Sishenchong (EX-HN1), and bilateral Hegu (LI-4), Neiguan (PC-6), Shenmen (HT-7), Taichong (LR-3), Sanyinjiao (SP-6), and Xuanzhong (GB-39) were selected for needling based on our previous studies on acupuncture for IA [17, 24]. All these acupuncture points were located according to the WHO Standard Acupuncture Point Locations in the Western Pacific Region. Stainless steel needles with 0.30 mm diameter and 40 mm length were inserted into the skin to a depth of 1.5-2.5 cm and twisted until the subjects felt deqi sensation and then retained in place for 30 min. The acupuncture treatment was administered every other day for a period of 40 days. To ensure the pure effect of acupuncture treatment, adolescents with IA were instructed not to take any other therapeutic intervention during the whole research. The IAT and the visual analogue scale (VAS) [25] were fulfilled by IA subjects to assess the symptom severity and the Internet craving, respectively, before and after the acupuncture treatment.

### 2.3. Resting-State MRI Data Acquisition

All fMRI images were acquired with a 3.0 T MRI scanner (GE Discovery MR 750, USA) with a standard 8-channel head coil. During scanning, participants were instructed to keep in supine position with eyes closed and nothing in mind. Participants wore ear plugs throughout the experiment to minimize the noise from the MRI scanner. A head holder was also used to restrict the displacement of the head. Functional images were collected using a standard Echo Planar Imaging sequence with the following parameters: repetition time = 2000 ms, echo time = 30 ms, flip angle = 90°, field of view = 24 × 24 cm^2^, image matrix = 64 × 64, and voxel size = 3.75 × 3.75 × 4.4 mm^3^. Two hundred and fifty-five volumes of image were obtained, and each of the volumes included thirty-five axial slices.

Two separate resting-state fMRI scans were performed on IA individuals, one within 3 days before the acupuncture treatment and the other after the treatment course within 3 days. The HCs participated in one fMRI scan as control.

### 2.4. Resting-State fMRI Data Processing

The data preprocessing was conducted using the neuroscience information toolbox (NIT, http://www.neuro.uestc.edu.cn/NIT.html), which is based on Statistical Parametric Mapping (SPM8, https://www.fil.ion.ucl.ac.uk/spm/). Prior to preprocessing, the first 5 volumes of all datasets were discarded to eliminate the interference of instability in the initial signals. Afterwards, slice timing was performed to correct the time delay between slices by aligning to the first image of each session. Spatial realignment was subsequently conducted to correct the head motion. Subjects with more than 2° rotation or more than 2 mm displacement were excluded in the current study. After spatial normalization based on the Montreal Neurological Institute (MNI) template with a resolution of 3 × 3 × 3 mm^3^, we regressed out nuisance signals, including 24 head motion parameters and signals from white matter and cerebral spinal fluid (CSF). Then, we smoothed the maps with a Gaussian kernel of 6 mm. Finally, the functional images were filtered (bandpass, 0.01-0.08 Hz) to reduce the interference of low-frequency drift and high-frequency noise.

### 2.5. Resting-State rsFC Analysis

Using Resting-State fMRI Data Analysis Toolkit (REST, http://www.restfmri.net/forum/REST_V1.8), two different rsFC analyses were conducted: seed-to-voxel and ROI-to-ROI analyses. For both methods, the mean BOLD temporal series of ROIs were extracted initially. Following that, Pearson's correlation analyses were performed between the time-varying signals of each seed and those of each voxel in the whole brain (seed-to-voxels)/every other seed (ROI-to-ROI). Finally, Fisher transformation was applied to convert the resulting correlation coefficients into normally distributed scores.

In seed-to-voxel analysis, VS seeds were identified as the superior ventral striatum (VSs; ±13, 15, 9), inferior ventral striatum (VSi; ±9, 9, -8), and ventral rostral putamen (VRP; ±20, 12, -3) (6 mm-radius sphere centered at the coordinates) according to the previous publications [26, 27]. The regions exhibiting altered rsFC to VS were selected as seeds in the following ROI-to-ROI analysis.

### 2.6. Statistical Analysis

#### 2.6.1. Analysis of Demographic and Clinical Data

Demographic and clinical characteristics were compared by SPSS 18. A two-sample *t*-test was performed to compare the continuous variable (e.g., age, IAT score) differences between HCs and IA individuals before treatment, while a paired  *t*-test was conducted within the IA group between post- and pretreatment. Categorical variable (e.g., gender) was analyzed by the chi-square test. A *p* value less than 0.05 was considered to be statistically significant.

#### 2.6.2. Analysis of rsFC

To compare the differences in the seed-to-voxel and ROI-to-ROI rsFC between HCs and IA individuals, a two-sample *t*-test was performed at baseline. A paired *t*-test was implemented to estimate rsFC changes between ROIs after treatment in IA individuals. The statistical significance for rsFC analysis was set at *p* < 0.05 (FDR corrected and cluster size > 621 mm^3^).

#### 2.6.3. Correlation Analysis between rsFC Changes and Clinical Improvement

To investigate the potential association between changes in functional communication and clinical improvement in IA individuals after treatment, we performed a partial correlation analysis between the clinical score improvement (score change/baseline score) and the connectivity strength changes (rsFC change/baseline rsFC), with gender, age, and years of education as covariates. The significance threshold was set to *p* < 0.05.

## 3. Results

A total of 60 subjects (30 IA subjects and 30 HCs) were recruited in our study initially. All 30 IA subjects participated in the first fMRI scan, but only 27 of them went on the second scan. There were 3 IA subjects who failed to participate in the second fMRI scan due to scheduling conflicts. All HCs finished the fMRI scan. No subject was excluded due to incomplete scan or excessive head movement. Thus, all analyses in our study were based on these 57 subjects (27 IA subjects and 30 HCs). Thirteen of the 27 IA subjects were included in our prior article investigating the abnormal functional connectivity density in IA [28] and further consented to participate in the present study to explore the modulation effect of acupuncture on IA.

### 3.1. Demographic Characteristics and Treatment Response

As shown in Table 1, no significant intergroup difference was observed in age, gender, or years of education at baseline (*p* > 0.05). A total of 27 IA individuals finished the acupuncture course as planned. After 40 days of acupuncture treatment, the IA group showed a significant decrease in IAT and VAS scores (*p* < 0.001).

### 3.2. Resting-State fMRI Results

#### 3.2.1. Seed-to-Voxel Analysis

**Figure 1:**
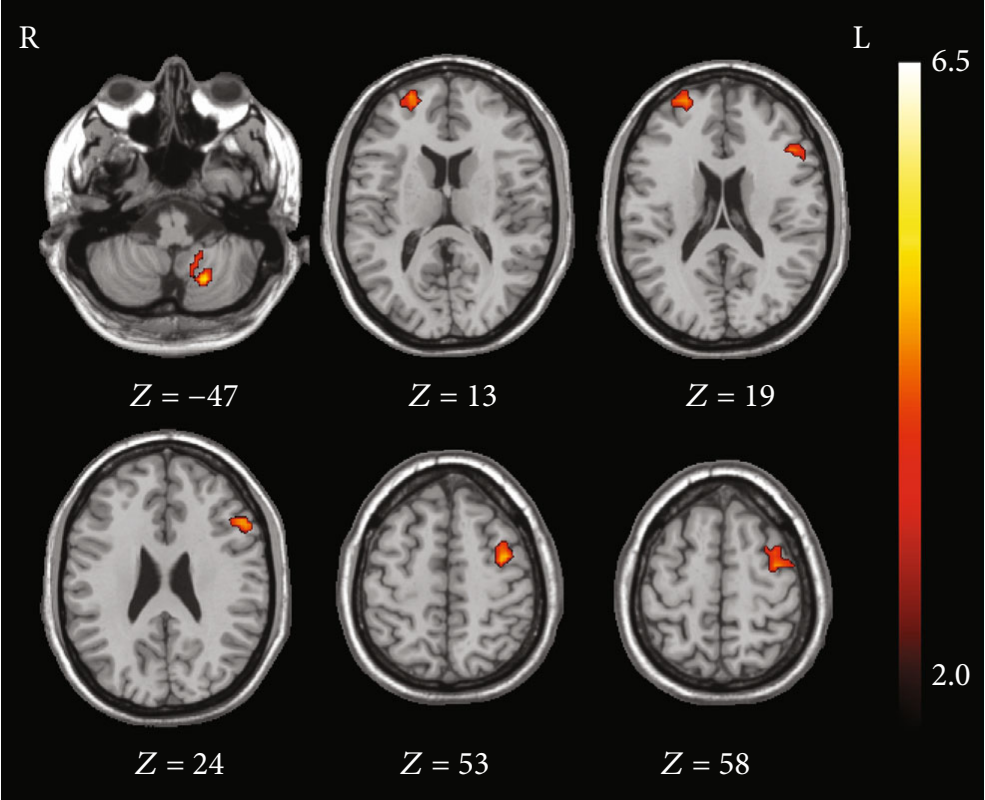
Significant intergroup differences in seed-to-voxel resting-state functional connectivity of the right ventral rostral striatum between the Internet addiction individuals and healthy control subjects. Compared with healthy control subjects, individuals with IA exhibited increased resting-state functional connectivity of the right ventral rostral putamen in the left cerebellum, premotor cortex, orbitofrontal cortex, and right ventromedial prefrontal cortex.

As shown in Table 2 and Figure 1, IA individuals exhibited enhanced rsFC of the right VRP with the left premotor cortex (PMC), orbitofrontal cortex (OFC), cerebellum, and right ventromedial prefrontal cortex (vmPFC) in comparison to HCs (*p* < 0.05, FDR corrected, cluster size > 621 mm^3^). No significant connectivity alteration was found in the left VRP or bilateral VSi or VSs.

#### 3.2.2. ROI-to-ROI Analysis

**Figure 2:**
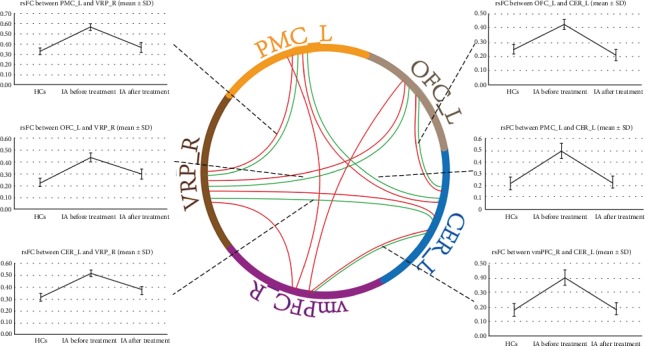
ROI-to-ROI rsFC analysis between IA subjects and HCs at baseline and between pre- and posttreatment in IA individuals. (1) Red solid line: compared with HCs, IA adolescents exhibited increased rsFC of the right VRP with the left PMC, OFC, cerebellum, and right vmPFC, as well as enhanced rsFC of the left cerebellum with the left PMC, OFC, and right vmPFC at baseline. Higher rsFC of the right vmPFC with the left OFC and PMC were also observed. (2) Blue solid line: after treatment, the rsFC of the right VRP with the left PMC, OFC, and cerebellum in IA subjects were reduced, as well as the rsFC of the left cerebellum with the left PMC, OFC, and right vmPFC. rsFC: resting-state functional connectivity; IA: Internet addiction; HCs: healthy control subjects; L: left; R: right; VRP: ventral rostral putamen; PMC: premotor cortex; OFC: orbitofrontal cortex; vmPFC: ventromedial prefrontal cortex; CER: cerebellum.

Besides the altered rsFC of the right VRP, the rsFC of the left cerebellum with the left PMC, OFC, and right vmPFC in the IA group were found increased at baseline in comparison to HCs (*p* < 0.05, FDR corrected). The increased rsFC of the right vmPFC with the left OFC and PMC were also observed in individuals with IA (*p* < 0.05, FDR corrected) (Figure 2).

After 40 days of acupuncture treatment, the rsFC of the right VRP with the left PMC, OFC, and cerebellum in IA subjects were reduced, as well as the rsFC of the left cerebellum with the left PMC, OFC, and right vmPFC (*p* < 0.05, FDR corrected) (Figure 2).

### 3.3. Correlation Analysis

**Figure 3:**
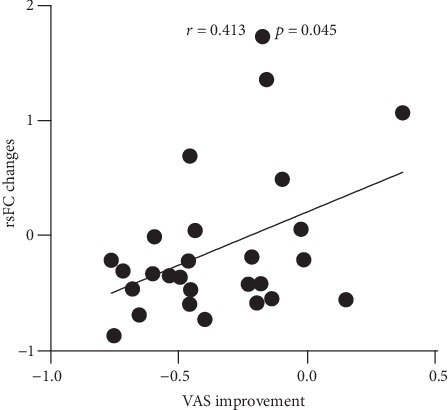
Partial correlation between rsFC changes and clinical improvement after treatment. After controlling for gender, age, and years of education, the rsFC change between the right VRP and left cerebellum in the IA group was positively correlated with the VAS score improvement after treatment. rsFC: resting-state functional connectivity; IA: Internet addiction; VRP: ventral rostral putamen; VAS: visual analogue scale.

After controlling for gender, age, and years of education, the change in rsFC strength between the right VRP and left cerebellum in the IA group was found positively correlated with the VAS score improvement after acupuncture treatment (*r* = 0.413, *p* = 0.045) (Figure 3).

## 4. Discussion

Defined as a behavioral addiction, IA has been considered a global public health and social problem, due to its overlapping manifestations with substance addiction and high prevalence all over the world [1]. Acupuncture, an effective therapy on neuropsychiatric disorders, is widely applied on the treatment of IA [13, 15, 24]. Nevertheless, the mechanisms of acupuncture on IA are far from clarified. In the current study, we used seed-to-voxel and ROI-to-ROI rsFC analyses to investigate the abnormal functional interactivity in IA individuals and to explore the modulation effect of acupuncture. The results showed that the IA individuals exhibited enhanced rsFC of the right VRP with the left OFC, PMC, cerebellum, and right vmPFC, as well as increased rsFC of the left cerebellum with the left OFC, PMC, and right vmPFC at baseline. The strengthened rsFC of the right vmPFC with the left OFC and PMC were also observed. All these regions were involved in the two main systems accounting for addiction in line with the I-RISA model, reward system and habit system, implicating the abnormal reward and habit processes in IA individuals [7]. After 40 days of acupuncture, the increased rsFC of the right VRP with the left OFC, PMC, and cerebellum were normalized, as well as the enhanced rsFC of the left cerebellum with the left OFC, PMC, and right vmPFC. The rsFC change between the right VRP and the left cerebellum in IA individuals was positively correlated with the Internet craving alleviation after acupuncture, according to the following correlation analysis. We believe these findings might elucidate the underlying neurophysiological mechanisms of acupuncture on IA partially.

### 4.1. Increased rsFC of Reward System in IA

As expected, increased functional interactivity of the reward system in IA was observed in our study. The IA individuals exhibited enhanced rsFC among the left OFC, vmPFC, and right VRP; all of which were core reward regions [29, 30]. The VS, receiving dopaminergic projection from ventral tegmental area (VTA) and projecting to prefrontal cortex, was proposed as a central pivot in the reward system [31]. Both OFC and vmPFC were involved in value assessment and decision-making and therefore played an important role in reward processing [30]. The reciprocal projection found between the VS and the other two areas implicated the interactive network among these reward regions [31–33]. Multiple meta-analyses of task-fMRI research further confirmed the distinct coupled network of these areas during reward processing tasks [34, 35], which was suggested as a key critical element in substance addiction. In IA, greater responses in VS, vmPFC, and OFC in reward outcome processing were also revealed by previous research [36]. The present study further revealed the higher rsFC among VS, OFC, and vmPFC, implicating the excessive function of reward in IA.

The cerebellum, well known for the function of motor control, was conventionally considered as an exclusive motor center. However, increasing evidence for activation of the cerebellum in various nonmotor processes has been found in recent studies, including emotion, working memory, in particular reward, and reinforcement learning [37]. Through virus transneuronal tracers, the reciprocal connections between the cerebellum and striatum and between the cerebellum and prefrontal areas were revealed [38, 39]. The VS-cerebellar loop was thought to be involved in reward-related learning and drug craving [40, 41], and altered function in this loop has been observed across addiction studies irrespective of the drug of abuse, including cocaine, heroin, and morphine [42–44]. Analogous results were observed in behavioral addictions, including IA [45]. The abnormal OFC/vmPFC-cerebellar loop was also observed in various substance addictions, which was thought to contribute to craving for addictive substance [46, 47]. As to IA, altered microstructure and function in the cerebellum, OFC, and vmPFC were also observed by previous neuroimaging studies [22, 48, 49]. Accordingly, the enhanced rsFC of the cerebellum with VS, OFC, and vmPFC revealed in our study might implicate the excessive Internet craving in IA individuals.

### 4.2. Increased rsFC of Habit System in IA

The overconnected VRP-PMC loop, concluded as a habit system in the I-RISA model [7], was revealed in our study. The habit system, responsible for generation of automatized behavior to adapt circumstances, was involved in transition from response-outcome goal-directed behavior to response-stimulus habitual behavior [50]. The hyperactive habit system in addiction was considered to account for the transition from voluntary to habitual and compulsive drug seeking [50].

The putamen, responsible for habit learning, was found dysfunctional in many psychotic disorders with altered habitual behaviors, in particular in addiction [51, 52]. Until recently, the PMC had been known as a specialized motor area. Nevertheless, mounting evidence associating the PMC lesions with impaired learning and other cognitive functions was observed in both humans and nonhuman primates [53, 54]. Hyperactivity of PMC was further observed in habitual behavior studies [55]. Recent neurophysiology research finally proved the critical role of PMC in transformation memory into a particular sequence, namely, habit [56]. Robust evidence for distinct projection between putamen and PMC was revealed by prior neuroarchitectural studies [57]. Furthermore, the white matter tract strength between PMC and putamen was presumed as a predictor of habitual performance [58]. In substance addicts, the PMC-putamen loop was revealed hyperactive when viewing drug-related images, which was presumed responsible for the automatized behavioral responsiveness to addicted cues [59]. Similarly, the strengthened rsFC between VRP and PMC revealed in our study might represent the potential mechanisms of the habitual Internet use in IA individuals.

The cerebellum, involved in rapid and automatic behavioral responses, was suggested as a key node in the process underlying habit formation [60]. As a result, cerebellar lesions might lead to failure of developing habits or new skills in both humans and rodents [61, 62]. As to addiction, over responsiveness of the cerebellum was found in a variety of substance dependence, associated with the habitual and compulsive use of drug [60]. Additionally, reciprocal projections between the cerebellum and PMC were revealed by virus transneuronal tracers [41]. Furthermore, the greater activity in the PMC-cerebellar loop was demonstrated in substance dependence [63], implicating the overactivation of habitual process. Analogous results were also observed in IA [64]. In our study, the increased rsFC between the PMC and cerebellum was observed, which further illustrated the possible mechanisms underlying the compulsive use of Internet in IA individuals.

### 4.3. The Modulation Effect of Acupuncture on Abnormal rsFC in IA

Acupuncture, a traditional nondrug therapy, was widely applied in a variety of substance addictions. Previous clinical studies have proved the effect of acupuncture on alleviating craving and addicted symptoms in substance dependence [65]. Similar to those in substance addiction, the decreased IAT and VAS scores were observed after 40 days of acupuncture treatment in our study, demonstrating the analogous effect of acupuncture in IA.

Previous studies have revealed the normalization of structure and function of reward regions in substance addicts by acupuncture [66, 67], indicating the effect of acupuncture in modulation of reward function. Similarly, our neuroimaging results showed that the increased rsFC of the right VRP with the left OFC and cerebellum was reduced after acupuncture treatment, as well as the enhanced rsFC of the left cerebellum with the left OFC and right vmPFC, implicating the normalizing effect of acupuncture on hyperactive reward process in IA. Furthermore, the normalization of overconnectivity of the left PMC with the left cerebellum and right VRP was also observed after treatment, representing the modulation effect of acupuncture on hyperactive habitual function in IA adolescents. To our knowledge, the present study was the first to investigate the modulation effect of acupuncture on rsFC in IA individuals. In addition, the correlation analysis revealed the association between the alleviation of Internet craving and the change in rsFC of the right VRP with the left cerebellum, further linking the improvement of symptom to recovery of reward function.

### 4.4. Limitations

There are still several issues to be addressed. First of all, one more fMRI scan should have been conducted for HCs to exclude the time effect, though FC of HCs was thought reliable in a long interval in some previous studies [68, 69]. Secondly, assessment of the follow-up effect of acupuncture was lacked in the current study, which should be considered in future studies. In addition, there is no task to measure the stimulus-response behaviors in IA, which restricts our understanding of contribution of the acupuncture to the alleviation of habitual use of Internet. Furthermore, a sham acupuncture group is needed to make conclusions more comparable and convincing. Lastly, all subjects in this study are recruited from college students that might limit the generalization of our findings.

## 5. Conclusions

In conclusion, IA was associated with increased functional connectivity of reward and habit systems. Our findings demonstrated the modulation effect of acupuncture on the hyperactive reward and habit systems, which might partly interpret the underlying neurophysiology mechanisms of acupuncture treatment on IA.

## Figures and Tables

**Table 1:** Demographic characteristics and clinical information of the participants.

	Healthy control group (*n* = 30)	IA group pretreatment (*n* = 27)	IA group posttreatment (*n* = 27)
	m ± SD	m ± SD	m ± SD
Age (years)	21.73 ± 2.08	22.44 ± 2.42^#^	22.44 ± 2.42
Gender (male/female)	22/8	20/7^#^	20/7
Education (years)	15.77 ± 1.82	15.80 ± 2.17^#^	15.80 ± 2.17
Internet addiction test scale	/	65.26 ± 14.36	45.85 ± 12.37^∗^
Visual analogue scale	/	7.30 ± 1.24	4.67 ± 1.94^∗^

^#^Comparison between IA individuals and HC subjects at baseline, *p* > 0.05. ^∗^Comparison in the IA group between post- and pretreatment, *p* < 0.001.

**Table 2:** Increased resting-state functional connectivity of the right VRP in the IA group as compared with HCs.

Regions	MNI coordinates			Peak *T* value	Cluster voxels
	*X*	*Y*	*Z*		
CER_L	-21	-66	-48	5.24	50
PMC_L	-36	-6	54	5.11	81
vmPFC_R	24	57	16	4.93	68
OFC_L	-50	16	21	3.74	48

VRP: ventral rostral putamen; IA: Internet addiction; HCs: healthy controls; MNI: Montreal Neurological Institute; L: left; R: right; PMC: premotor cortex; OFC: orbitofrontal cortex; vmPFC: ventromedial prefrontal cortex; CER: cerebellum.

## Data Availability

The fMRI data used to support the findings of this study are available from the corresponding author upon request.
